# Cathepsin Z as a novel potential biomarker for osteoporosis

**DOI:** 10.1038/s41598-019-46068-0

**Published:** 2019-07-05

**Authors:** Ayed A. Dera, Lakshminarayan Ranganath, Roger Barraclough, Sobhan Vinjamuri, Sandra Hamill, Dong L. Barraclough

**Affiliations:** 1 Department of Musculoskeletal Biology, Institute of Ageing and Chronic Disease, University of Liverpool, The William Henry Duncan Building, 6 West Derby Street, Liverpool, L7 8TX United Kingdom; 2 Department of Clinical Biochemistry and Metabolic Medicine, The Royal Liverpool and Broadgreen University Hospital NHS Trust, Prescot Street, Liverpool, L7 8XP United Kingdom; 3 Department of Biochemistry, Institute of Integrative Biology, University of Liverpool, Biosciences Building, Crown Street, Liverpool, L69 7ZB United Kingdom; 4 Department of Nuclear Medicine, The Royal Liverpool and Broadgreen University Hospital NHS Trust, Prescot Street, Liverpool, L7 8XP United Kingdom; 5 Present Address: Department of Clinical Laboratory Sciences, College of Applied Medical Sciences and Research Centre of Advanced Materials, King Khalid University, Abha, Saudi Arabia

**Keywords:** Diagnostic markers, Biomarkers

## Abstract

Osteoporosis, one of the most prevalent chronic ageing-related bone diseases, often goes undetected until the first fragility fracture occurs, causing patient suffering and cost to health/social care services. Osteoporosis arises from imbalanced activity of osteoclasts and osteoblasts. Since these cell lineages produce the protease, cathepsin Z, the aim of this study was to investigate whether altered cathepsin Z mRNA levels are associated with osteoporosis in clinical samples. Cathepsin Z mRNA in human peripheral blood mononuclear cells was significantly differentially-expressed among non-osteoporotic controls, osteopenia and osteoporosis patients (*p* < 0.0001) and in female osteoporosis patients over the age of 50 years (*P* = 0.0016). Cathepsin Z mRNA level strongly correlated with low bone mineral density (BMD) (g/cm^2^), lumbar spine L2-L4 and femoral neck (*T*-scores) (*P* = 0.0149, 0.0002 and 0.0139, respectively). Importantly, cathepsin Z mRNA was significantly associated with fragility fracture in osteoporosis patients (*P* = 0.0018). The levels of cathepsin Z mRNA were not significantly higher in patients with chronic inflammatory disorders in these two groups compared to those without (*P* = 0.774 and 0.666, respectively). ROC analysis showed that cathepsin Z mRNA has strong diagnostic value for osteoporosis and osteoporotic fracture. The results show for the first time that cathepsin Z could be a future diagnostic biomarker for osteoporosis including female osteoporosis patients over the age of 50 years.

## Introduction

Osteoporosis is one of most common chronic ageing-related bone diseases in the world^1^, and is often, but not exclusively, associated with the reduced bone density in post-menopausal women, arising from reduced estrogen levels^2^. Osteoporosis often goes undetected until the first fragility fracture occurs, which causes unnecessary suffering to the patient and cost to the health service^1^. Thus, there is a need to identify biomarkers that could be exploited, in the future, for a clinical test that could indicate the presence of osteoporosis before fragility fracture occurs.

Osteoporosis is thought to arise from an imbalance between the activity of bone degrading osteoclasts, derived from monocyte lineages of immune cells^3^ and bone-synthesising osteoblasts derived from mesenchymal stem cells^4^. The lysosomal cysteine protease, cathepsin Z, believed to be encoded by the same mRNA sequence as cathepsin X (NCBI reference sequence NM_001336), is highly expressed in cells of the immune system. In particular, it is expressed in T-lymphocytes^5^ and monocytes^6^ the latter being precursors of osteoclasts^7^, which produce the related protease, cathepsin K that has been shown to be required for bone resorption^8^. Both T-lymphocytes and monocytes are components of the peripheral blood mononuclear cell (PBMC) fraction that can be isolated from whole blood^9^. In addition, cathepsin Z protein has been reported to be secreted by human osteoblasts^10^ and thus cathepsin Z appears to be produced in cells of both osteoclast and osteoblast lineages.

Alterations in the expression of the cathepsin Z gene have been linked, bioinformatically, to fragility fractures in cancer patients in association with reduced levels of circulating estrogen^11^. A genome wide association study into the genetic factors that may be associated with fragility fractures arising from the treatment of estrogen-receptor-positive breast cancer patients with aromatase inhibitor, identified a single-nucleotide polymorphism in or near a 3-gene cluster on chromosome 20 containing the cathepsin Z gene. This polymorphism was shown to be associated with the occurrence of fragility fractures^11^. Using an osteoblast cell culture model system, the estrogen-receptor dependence of cathepsin Z gene expression was demonstrated in these cells. The polymorphism not only affected the expression of the cathepsin Z gene, but experimental knockdown of cathepsin Z levels affected the expression of osteoporosis-associated genes^11^, suggesting an association between cathepsin Z expression, fragility fracture and the expression of osteoporosis-related genes in an estrogen-reduced, clinical situation. However, the direct effect of cathepsin Z on other cell activities was not studied.

That cathepsin Z is found in both osteoblast and osteoclast lineages of cells, the two cell types predominantly dysregulated in osteoporosis, raises the possibility that the occurrence of osteoporosis in human subjects might be associated with changes in cathepsin Z expression in these cells. In the present experiments, it is shown that levels of cathepsin Z mRNA in clinical samples of peripheral blood mononuclear cells are significantly associated with osteoporosis, low bone mineral density and occurrence of fragility fractures.

## Results

### Characteristics of clinical samples

A total of 88 participants were recruited, 71 participants were female and 17 were male (Table 1). Bone mineral density (BMD) and *T*-Score were obtained following standard clinical procedures. Participants were categorised into three groups based on their *T*-Scores: a non-osteoporotic control group having *T* score ≥−1.0, an osteopenia group with *T* score <−1.0 and >−2.5 and an osteoporosis group with *T* score ≤−2.5 (Table 1). Nineteen percent of the participants were non-osteoporotic (14 female and 3 male), fifty five percent of the participants were osteopenia (38 female and 10 male) and twenty six percent were suffering from osteoporosis (19 female and 4 male). The average age of participants was 56 ± 18.9 years for non-osteoporotic control, 65 ± 9.7 years old for osteopenia and 69 ± 13.2 years old for osteoporosis patients. 88% of participants were over 50 years old, comprising 82% female and 18% male. For the over-50-year-old participants, 13% were non-osteoporotic controls (mean age, 70 ± 5.5 years), 58% were osteopenia (mean age 67 ± 7.8 years) and 29% were osteoporosis patients (mean age 71 ± 8.2 years).

**Table 1:** Summary of Characteristics of Clinical Samples.

Clinical category	Non-osteoporotic control	Osteopenia	Osteoporosis
**Number of participants**			
Female	14	38	19
Male	3	10	4
Total	17	48	23
**Participants age (mean ± SD years) Female**			
Female	58** ± **17.9	64** ± **8.8	69** ± **13.9
Male	47** ± **24.6	67** ± **12.9	67** ± **10.2
Total	56** ± **18.9	65** ± **9.7	69** ± **13.2
**Number of participants >50 years of age**			
Female	9	36	18
Male	1	9	4
Total	10	45	22
**Participants >50 years of age (mean ± SD years)**			
Female	70** ± **5.6	66** ± **7.5	72** ± **7.8
Male	75** ± **0	70** ± **8.4	67** ± **10.2
Total	70** ± **5.5	67** ± **7.8	71** ± **8.2
**Number of patients with other diseases (chronic inflammatory diseases)**			
Female	0	9 (9)	5 (3)
Male	0	3 (2)	2 (1)
Total*	0	12 (11)	7 (4)
**Number of patients treated with Bisphosphonate or Denosumab**			
Female	0	5	9
Male	0	3	0
Total**	0	8	9
**Number of participants with fracture history**			
Female	3	20	8
Male	1	4	1
Total	4	24	9
BMD (g/cm^2^) (Mean** ± **SD)	0.964** ± **0.108	0.841** ± **0.107	0.710** ± **0.074
Lumbar 2–4 *T-*score (Mean** ± **SD)***	0.427	−1	−2.604
Femoral neck *T*-score (Mean** ± **SD)	−0.18	−1.285	−2.296

^*^Twelve osteopenia patients (nine female and three male) and seven osteoporosis patients (five female and two male) had other diseases including hypertension and type 2 diabetes and chronic inflammatory diseases: arthritis, asthma, coeliac disease, COPD, Crohn’s disease, osteoarthritis, rheumatoid arthritis or were being treated with steroids.

**Eight osteopenia (five female and three male) and seven female osteoporosis patients were being treated with bisphosphonate, such as Alendronate, Ibandronate, Risedronate and Zoledronic acid. Two female osteoporosis patients were treated with Denosumab.

***Patients were categorised as osteopenia or osteoporosis based on either a low femoral neck or lumbar 2–4 *T*-score, thus some osteopenia patients with low femoral neck scores had normal lumbar 2–4 *T* scores.

Eight osteopenia (5 female and 3 male) and seven female osteoporosis patients were being treated with bisphosphonate, such as Alendronate, Ibandronate, Risedronate and zoledronic acid. Two female osteoporosis patients were being treated with Denosumab.

### The levels of cathepsin Z mRNA in PBMCs of human subjects are associated with osteoporosis

**Figure 1:**
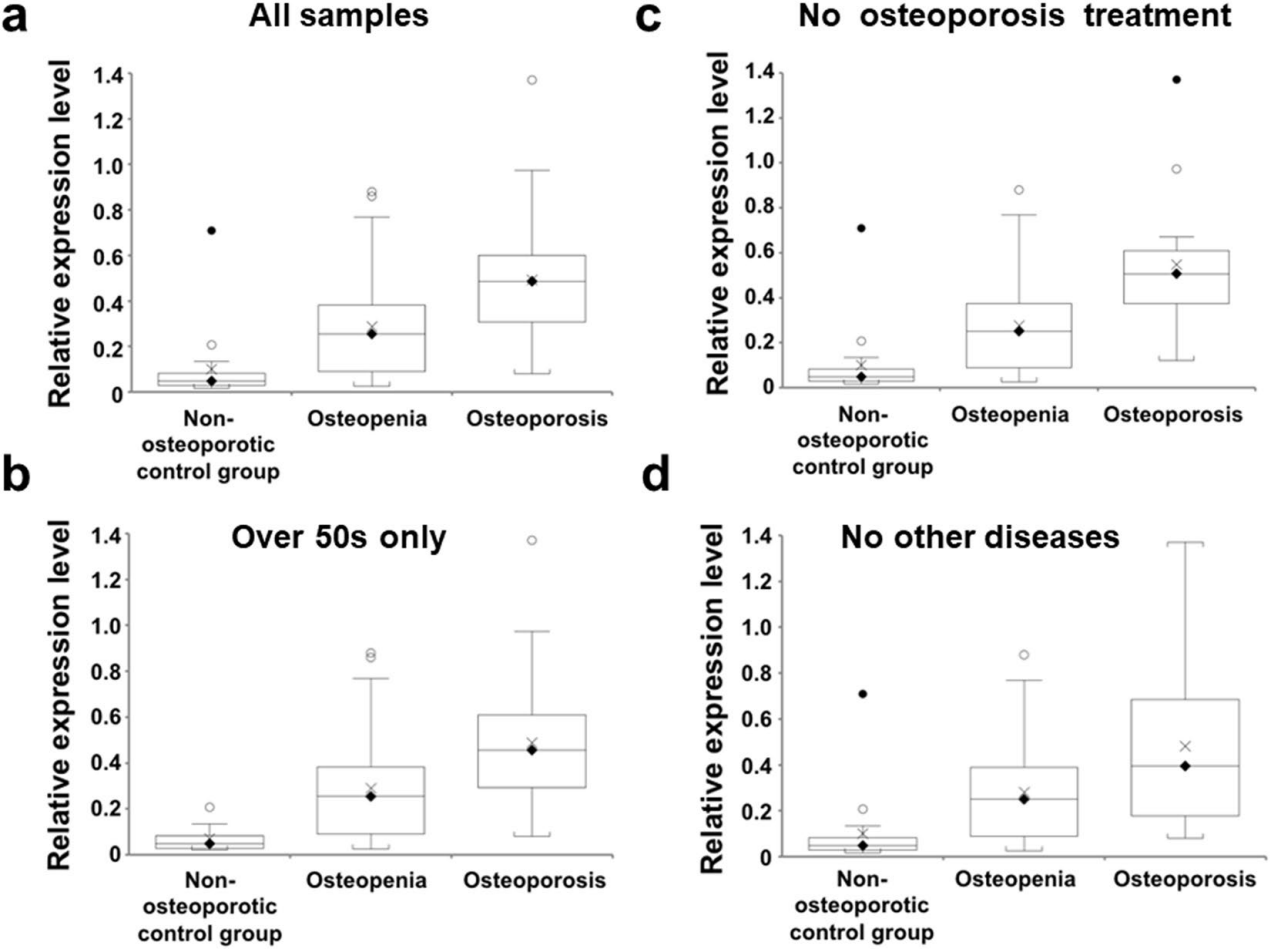
Association with osteoporosis of cathepsin Z mRNA levels in peripheral blood mononuclear cells. Panel (a) box and whisker plot shows significant differences in the levels of cathepsin Z mRNA between all non-osteoporotic controls, patients with osteopenia and patients with osteoporosis (*P* < 0.0001, ANOVA). Similar significant differences were evident when the analysis was confined to subjects who were over 50 years of age (*P* < 0.0001, ANOVA; panel b) or when those subjects who were receiving treatment for osteoporosis were excluded from the analysis (*P* < 0.0001, ANOVA; panel c) or when subjects who were suffering from other non-osteoporotic disorders were excluded from the analysis (*P* = 0.0002, ANOVA, panel d). On each box and whisker plot, the black diamond shows the median value, the cross shows the mean value, white and black circles denote outliers of 1.5 times and 3 times the interquartile range, respectively.

Cathepsin Z mRNA levels were measured by RT-qPCR in PBMCs from the non-osteoporotic control group, the osteopenia group and the osteoporosis group as shown in Fig. 1. There was a strongly-significant increase in cathepsin Z mRNA in the order: non-osteoporotic control group, osteopenia group and osteoporosis group (Fig. 1a) (one-way ANOVA (F(2,85) = 13.4, *P* < 0.0001; non-osteoporotic control group vs osteopenia, 95% CI = −0.32 to −0.053, *P* = 0.0067; non-osteoporotic control group vs osteoporosis, 95%CI = −0.543 to −0.24, *P* = < 0.0001; osteopenia vs osteoporosis, 95% CI = −0.325 to −0.084, *P* = 0.0011, post hoc Bonferroni correction).

The significant increases in cathepsin Z mRNA levels in osteopenia/osteoporosis were also evident when 11 participants who were below the age of 50 were excluded from the analysis (Fig. 1b) (one-way ANOVA (F(2,74) = 10.9), *P* < 0.0001; non-osteoporotic control group vs osteopenia, 95% CI = −0.39 to −0.051, *P* = 0.0113; non-osteoporotic control group vs osteoporosis, 95% CI = −0.604 to −0.234, *P* = < 0.0001; osteopenia vs osteoporosis, 95% CI = −0.324 to −0.072, *P* = 0.0025, post hoc Bonferroni correction).

The results were not affected when 17 participants (8 osteopenia and 9 osteoporosis) receiving treatment for osteoporosis were excluded from the analysis (Fig. 1c) (one-way ANOVA (F(2,68) = 14.7), *P* < 0.0001; non-osteoporotic control group vs osteopenia, 95% CI = −0.308 to −0.043, *P* = 0.01; non-osteoporotic control group vs osteoporosis, 95% CI = −0.611 to −0.282, *P* = < 0.0001; osteopenia vs osteoporosis, 95% CI = −0.413 to −0.129, *P* = 0.0003, post hoc Bonferroni correction); nor when 19 patients (12 osteopenia and 7 osteoporosis) with non-osteoporotic disorders were excluded (Fig. 1d) (one-way ANOVA (F(2,66) = 9.6, *P* = 0.0002; non-osteoporotic control group vs osteopenia, 95% CI = −0.325 to −0.032, *P* = 0.0175; non-osteoporotic control group vs osteoporosis, 95% CI = −0.554 to −0.207, *P* < 0.0001; osteopenia vs osteoporosis, 95% CI = −0.351 to −0.052, *P* = 0.0089, post-hoc Bonferroni correction), suggesting that all the sample results were not subject to bias from age, osteoporosis treatment or the presence of other diseases in this cohort.

### Cathepsin Z associated with osteoporosis in female patients over the age of 50

**Figure 2:**
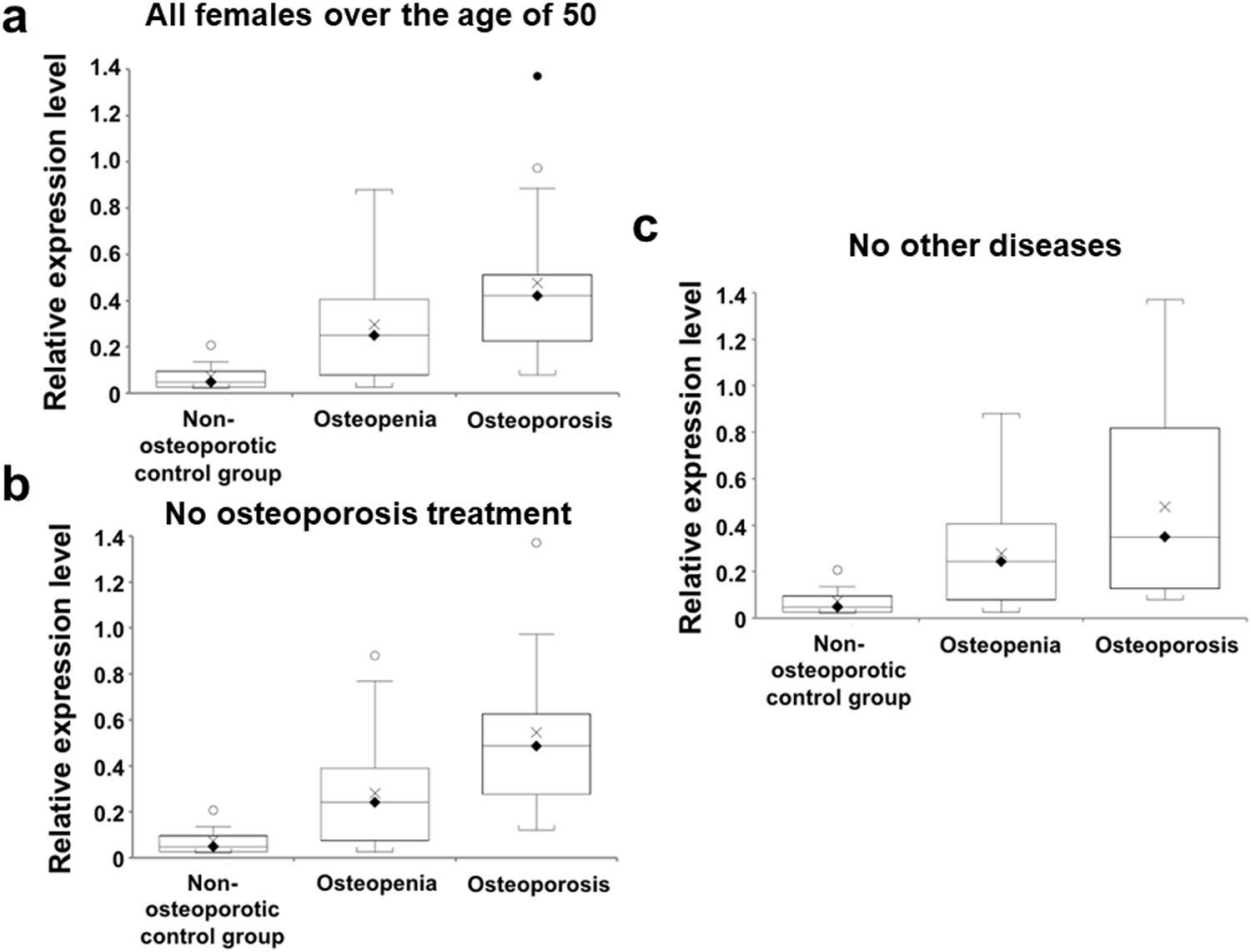
Association with osteoporosis of cathepsin Z mRNA levels in peripheral blood mononuclear cells of female participants over the age of 50. Panel a: box and whisker plot shows significant differences in the levels of cathepsin Z mRNA between female non-osteoporotic controls, patients with osteopenia and patients with osteoporosis all over the age of 50 (*P* = 0.0016, ANOVA). Similar, significant differences were evident when those subjects who were receiving treatment for osteoporosis were excluded from the analysis (*P* = 0.0012, ANOVA; panel b) or when subjects who were suffering from other non-osteoporotic disorders were excluded from the analysis (*P* = 0.0052, ANOVA, panel c). On each box and whisker plot, the black diamond shows the median value, the cross shows the mean value, white and black circles denote outliers of 1.5 times and 3 times the interquartile range, respectively.

The strong, significant increase in cathepsin Z mRNA levels in osteopenia and osteoporosis patients compared to the non-osteoporotic control group was also found when the analyses were confined to the 63 female participants over the age of 50-years as shown in Fig. 2 (one-way ANOVA (F(2,60) = 7.2), *P* = 0.0016; Fig. 2a, non-osteoporotic control group vs osteopenia, 95% CI = −0.422 to −0.027, *P* = 0.0266; non-osteoporotic control group vs osteoporosis, 95% CI = −0.621 to −0.188, *P* = 0.0004; osteopenia vs osteoporosis, 95% CI = −0.333 to −0.027, *P* = 0.0223, post-hoc Bonferroni correction) and the results were not affected when 14 patients (5 osteopenia and 9 osteoporosis) receiving treatment for osteoporosis were excluded as shown in Fig. 2b (one-way ANOVA (F(2,46) = 7.8), *P* = 0.0012; Fig. 2b, non-osteoporotic control group vs osteopenia, 95% CI = −0.402 to −0.013, *P* = 0.038; non-osteoporotic control group vs osteoporosis, 95% CI = −0.716 to −0.231, *P* = 0.0003; osteopenia vs osteoporosis, 95% CI = −0.461 to −0.072, *P* = 0.0084, post-hoc Bonferroni correction). When 13 patients (8 osteopenia and 5 osteoporosis) with non-osteoporotic disorders were excluded, there was no change to the overall result (Fig. 2c) (one-way ANOVA (F(2,47) = 5.89), *P* = 0.0052), or to the relationship between the osteoporosis and the non-osteoporotic control group (95% CI = −0.648 to −0.167, *P* = 0.0014, post-hoc Bonferroni correction) or to the relationship between the osteoporosis and osteopenia groups (95% CI = 0.014 to 0.386, *P* = 0.036, post-hoc Bonferroni correction). However, there was a slight loss of significance between the non-osteoporotic control group and osteopenia group (95% CI = −0.419 to 0.0056, *P* = 0.056).

### The levels of cathepsin Z mRNA and chronic inflammatory disorders in osteoporosis patients

Patients with chronic inflammatory diseases often suffer osteoporosis-like conditions^12^. It is possible that the observed increase in cathepsin Z mRNA level in participants’ PBMCs is a consequence of underlying chronic inflammatory disorders. However, there was no significant difference in cathepsin Z mRNA levels between osteopenia and osteoporosis patients who were also suffering from chronic inflammatory disorders and those that were not, either when all osteopenia/osteoporosis patients (Supplementary Fig. S1a) or only female osteopenia/osteoporosis patients over the age of 50 (Supplementary Fig. S1b) were included (all osteopenia and osteoporosis patients, 95% CI = −0.180 to 0.134, *P* = 0.774; female osteopenia and osteoporosis patients over the age of 50, 95% CI = −0.244 to 0.157, *P* = 0.666, Student’s t-test). However, it is also possible that the elevated levels of cathepsin Z mRNA could be a consequence of underlying chronic viral infections. The levels of the mRNA for γ interferon inducible protein, IFI16, were determined in the PBMCs of osteopenia and osteoporosis patients. IFI16 is associated with myeloid differentiation and is expressed in CD14+ monocytes^13^. However, unlike for cathepsin Z mRNA, there was no significant difference in IFI16 mRNA levels between non-osteoporotic controls, osteopenia and osteoporosis patients, either when all participants were included (Supplementary Fig. S2a) or when only females over the age of 50 (Supplementary Fig. S2b) were included (all participants: one-way ANOVA (F(2,84) = 2.05), *P* = 0.135; females over the age of 50: one-way ANOVA (F(2,60) = 0.612), *P* = 0.546). Furthermore, there was no significant difference in IFI16 mRNA levels between osteopenia and osteoporosis patients who were also suffering from chronic inflammatory disorders and those that were not, either when all osteopenia/osteoporosis patients (Supplementary Fig. S3a) or only female osteopenia/osteoporosis patients over the age of 50 (Supplementary Fig. S3b) were included (all osteopenia and osteoporosis patients, 95% CI = −0.032 to 0.097, *P* = 0.318; female osteopenia and osteoporosis patients over the age of 50, 95% CI = −0.075 to 0.088, *P* = 0.876, Student’s t-test). The results suggest that neither chronic inflammatory disorders nor chronic viral infections account for the observed increase in cathepsin Z mRNA levels in the osteopenia/osteoporosis patients compared to non-osteoporotic controls.

### Cathepsin Z associated with low bone mineral density and fracture

**Figure 3:**
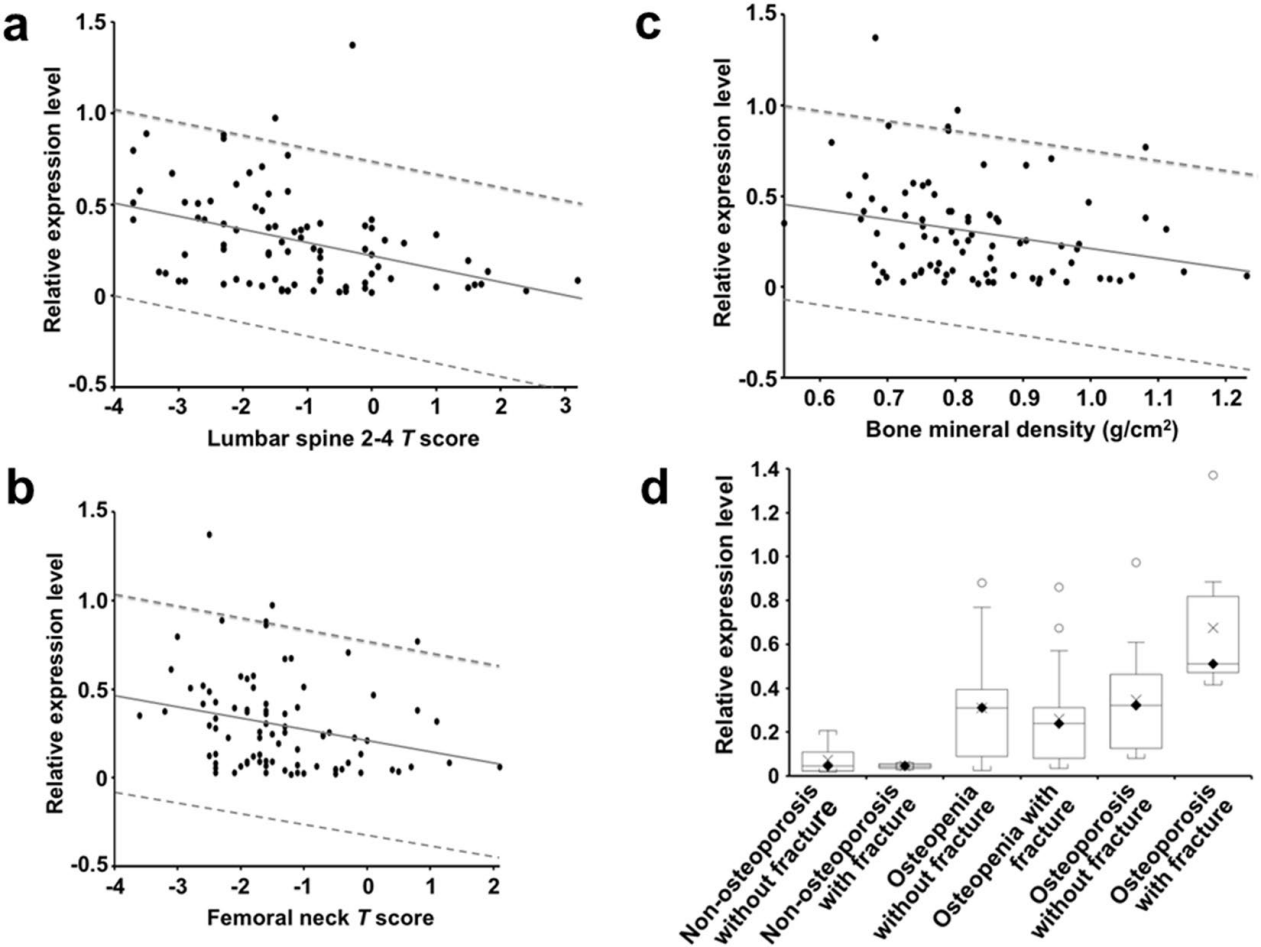
Relationship between the levels of cathepsin Z mRNA and bone mineral density and fracture history in all participants. The level of cathepsin Z mRNA significantly decreased with increasing *T*-score measured either in the lumbar spine (L2–4; *P* = 0.0002, linear regression; panel a) or at the femoral neck (*P* = 0.0139, linear regression; panel b). The level of cathepsin Z mRNA also significantly decreased with increasing bone mineral density BMD (g/cm^2^) (*P* = 0.0149, linear regression; panel c). The solid line denotes the linear regression line; the broken lines represent the 95% prediction intervals. Panel d: box and whisker plots show that while there was no significant difference in the level of cathepsin Z mRNA between non-osteoporotic controls with or without fracture (*P* = 0.851) or between osteopenia patients with or without fracture (*P* = 0.474), the cathepsin Z mRNA levels were significantly higher in osteoporosis patients with fracture than those without fracture (*P* = 0.0018). On the box and whisker plots, the black diamond shows the median value, the cross shows the mean value, white and black circles denote outliers 1.5-fold higher and 3-fold higher than the interquartile range, respectively.

Overall, the levels of cathepsin Z mRNA in isolated PBMCs were significantly inversely-associated with bone mineral density (Fig. 3): mean lumbar spine L2-L4 *T*-score (Fig. 3a) (y = −0.071645×+0.219589, r^2^ = 0.152, *P* = 0.0002, linear regression), significantly inversely with femoral neck *T*-score (Fig. 3b) (y = −0.065515×+0.213781, r^2^ = 0.0698, *P* = 0.0139) and significantly inversely associated with bone mineral density (BMD) (g/cm^2^) (Fig. 3c) (y = −0.553049×+0.760755; r^2^ = 0.07, P = 0.0149), strengthening the observed relationship between cathepsin Z mRNA levels and osteoporosis.

When the non-osteoporotic, osteopenia and osteoporosis patient groups were divided into those with and without previous fractures, there was no significant difference in the level of cathepsin Z mRNA between those with or without fracture for the non-osteoporotic control subjects (Fig. 3d) (95% CI = −0.254 to 0.306, *P* = 0.851, post-hoc Bonferroni correction) or for the osteopenia patients (95% CI = −0.086 to 0.184, *P* = 0.474, post-hoc Bonferroni correction), suggesting that cathepsin Z mRNA levels were not, per se, a consequence of the occurrence of the fracture in these groups. However, for the osteoporosis group of patients, those patients with previous fractures exhibited levels of cathepsin Z mRNA that were highly significantly higher than for those without previous fractures (Fig. 3d) (95% CI −0.529 to −0.126, *P* = 0.0018, post-hoc Bonferroni correction). Thus, an increase in cathepsin Z mRNA in patients’ PBMCs was associated with reduced bone density using three clinical measures, lumbar and femoral *T*-score and BMD and with fracture in osteoporosis patients.

### Diagnostic value of cathepsin Z mRNA

**Figure 4:**
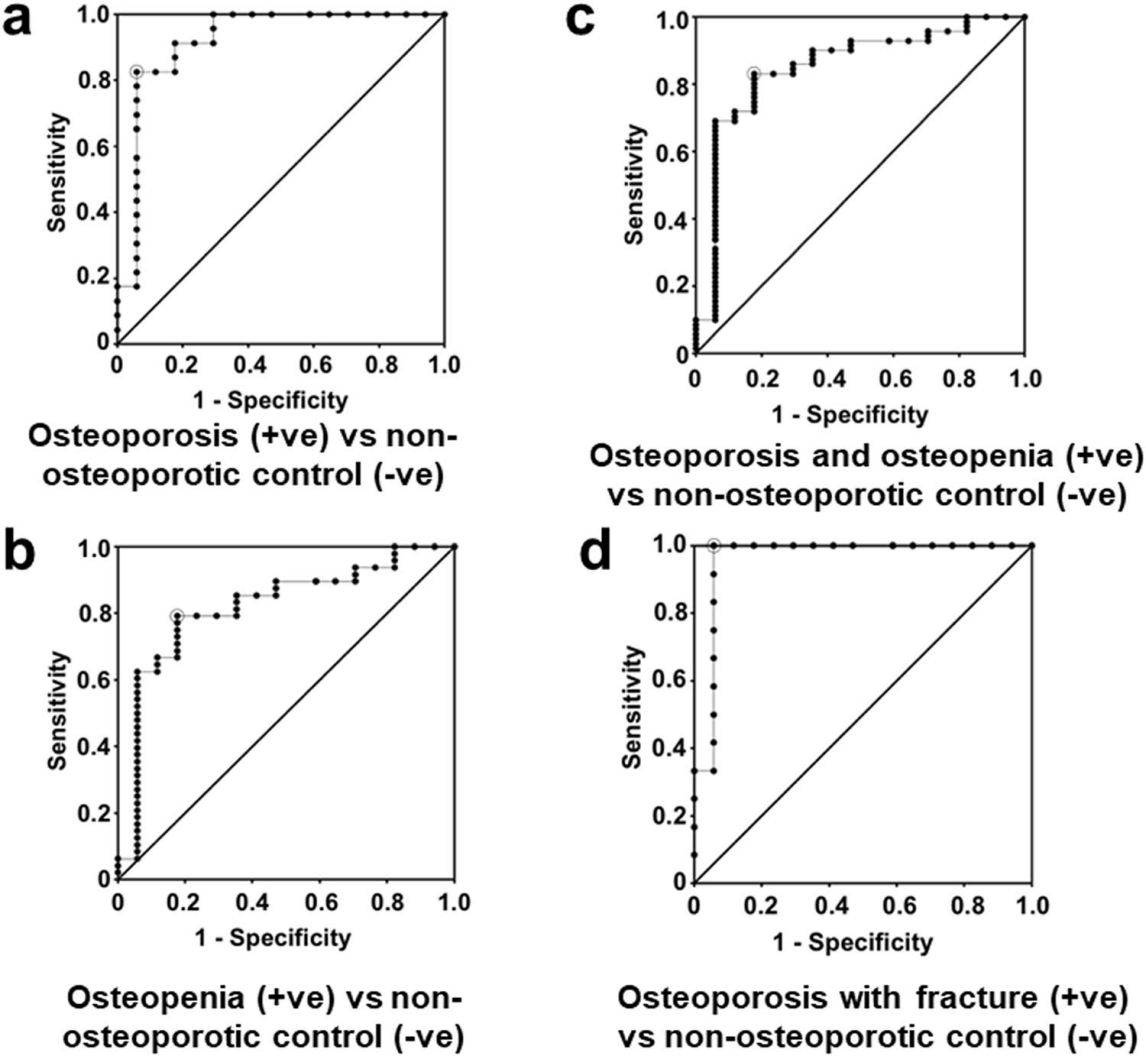
Diagnostic value of levels of cathepsin Z in PBMC for osteoporosis. Participants were divided into three groups based on their bone mineral density *T* scores (a non-osteoporotic control group having *T* scores ≥−1.0, an osteopenia group with *T* score <−1.0 and >−2.5 and an osteoporosis group with *T* score ≤−2.5). ROC curves are shown for the comparison between osteoporosis patients and non-osteoporotic controls (Panel a; AUC = 0.92, sensitivity, 82.6%, 95% CI = 61.2 to 95%; specificity, 94.1%, 95% CI = 71.3 to 99.9%)^22^, between osteopenia patients and non-osteoporotic controls (Panel b; AUC, 0.817; sensitivity, 79.2%, 95% CI = 65 to 89.5%; specificity, 82.4%, 95% CI = 56.6 to 96.2%^22^), between osteoporosis and osteopenia patients together and non-osteoporotic controls (Panel c; AUC = 0.851, sensitivity, 83.1%, 95% CI = 72.3 to 91%; specificity, 82.4%, 95% CI = 56.6 to 96.2%) and between non-osteoporotic controls and osteoporosis patients who had previously suffered a fracture (Panel d; AUC = 0.96, sensitivity 100%, 95% CI 73.5 to 100%; specificity, 94.1%, 95% CI 71.3 to 99.9%). AUC = Area under the curve. CI = Confidence interval. The large circle signifies the optimal cut-off.

To assess the potential diagnostic value of cathepsin Z mRNA in the PBMC for osteopenia/osteoporosis, receiver operator characteristics (ROC) curve analysis was performed. The diagnostic values of cathepsin Z mRNA in clinical samples for osteoporosis patients vs the non-osteoporotic control group was of high accuracy (Fig. 4a)^14^ (AUC, 0.92, sensitivity, 82.6%, 95% CI = 61.2 to 95%; specificity, 94.1%, 95% CI = 71.3 to 99.9%), stronger than the moderate accuracy for osteopenia vs non-osteoporotic control group (Fig. 4b) (AUC, 0.817; sensitivity, 79.2%, 95% CI = 65 to 89.5%; specificity, 82.4%, 95% CI = 56.6 to 96.2%) or for osteopenia and osteoporosis together vs non-osteoporotic control group (Fig. 4c) (AUC, 0.851; sensitivity, 83.1%, 95% CI = 72.3 to 91%; specificity, 82.4%, 95% CI = 56.6 to 96.2%). The diagnostic value of cathepsin Z mRNA in the osteoporosis patients that had experienced a previous fracture was even stronger than for the entire osteoporosis group (Fig. 4d) (AUC, 0.96; sensitivity 100%, 95% CI 73.5 to 100%; specificity, 94.1%, 95% CI 71.3 to 99.9%). These results suggest that cathepsin Z mRNA has strong diagnostic value for osteoporosis vs non-osteoporotic controls which might be associated with osteoporotic fracture, at least in this patient group.

## Discussion

Cathepsin Z mRNA has been shown for the first time to be significantly elevated in the PBMCs of patients with either osteopenia or osteoporosis compared to non-osteoporotic controls. For all participants, the mean ages of the non-osteoporotic control, osteopenia and osteoporosis groups were significantly different at 56, 65 and 69 years with the ages of the osteopenia and osteoporosis groups being significantly different from the non-osteoporotic control group (*P* = 0.025 and 0.0045 respectively, ANOVA post hoc Dunnett multiple comparison with a control). Thus, we cannot rule out entirely the possibility that cathepsin Z levels are, in part, a function of age for all participants. However, the increase in cathepsin Z mRNA level in osteopenia/osteoporosis was also observed when male and female participants over the age of 50, or female participants over the age of 50 were analysed. In these cases, the ages of the osteopenia and osteoporosis groups were not significantly different from the non-osteoporotic controls (male and female participants over the age of 50, osteopenia, *P* = 0.23 and osteoporosis *P* = 0.97; females over the age of 50, osteopenia, *P* = 0.185 and osteoporosis, *P* = 0.727, ANOVA post hoc Dunnett multiple comparison with a control). These observations suggest that at least for participants over the age of 50, age is not an important factor in the increase of cathepsin Z mRNA in osteopenia/osteoporosis.

That the PBMC preparations consist predominantly of immune cells raises the possibility that the observed osteoporosis-related increase in cathepsin Z mRNA reflects a change in the immune state of the osteopenia/osteoporosis patients relative to the normal controls. For example, osteoporosis could be a co-morbidity with chronic inflammatory disorders^12^, such as arthritis, COPD, coeliac disease. In the present group of participants, only 17% were recorded as suffering from chronic inflammatory conditions. However, in patients with osteopenia and osteoporosis, the cathepsin Z mRNA levels were not significantly different between those with and those without inflammatory disorders (*P* = 0.774). The results suggest that the increase in cathepsin Z mRNA in patients’ PBMCs was independent of chronic inflammation, at least in this group of patients. Similarly, no evidence was found for cathepsin Z mRNA levels being associated with virally-induced inflammation via the γ interferon pathway, although a more direct assessment of chronic inflammation, by directly assaying the blood levels of inflammatory cytokines, such as tumour necrosis factor (TNF alpha)^15^ would help to confirm these findings.

Cathepsin Z mRNA was measured in PBMC preparations from human subjects. Such preparations contain many types of circulating cells of the immune system, including both B and T lymphocytes^9^, dendritic cells^6^ and CD14+, CD16− monocytes^7^, some of the latter being precursors of osteoclasts^7^. Cathepsin Z mRNA/protein has been reported to be present at varying levels in all these cell types^5,6^. Thus, in the present study, the precise cell type that is the source of the elevated cathepsin Z mRNA is unknown. It has been reported, using PBMC preparations, that the mature form of cathepsin Z interacts with lymphocyte function-associated antigen-1 (LFA-1), a β-2-integrin on the surface of T lymphocytes^16^. Using specific cathepsin Z inhibitors, this interaction has been shown to alter T lymphocyte behaviour, for example, increasing activated proliferation rates in PBMCs^16^ and migration, invasion and aggregation using a cultured T cell line^5^. Cathepsin Z can also alter the behaviour of differentiated monocytes, by interacting with the Mac-1 β2-integrin^17^. Thus, the presence of elevated levels of cathepsin Z mRNA/protein in the PBMC cells of osteoporosis patients, might be associated with altered behaviour of monocytes and T lymphocytes. Whilst monocytes are the precursors of osteoclasts^7^, the role of T lymphocytes and the various subtypes in osteoporosis has been recognised, but is not yet fully understood^18^.

Irrespective of the biological effects of cathepsin Z, it has now been shown for the first time here that the increase in cathepsin Z mRNA in isolated PBMC fractions is very strongly diagnostic for osteoporosis, at least in the patient group studied. Overall, using the optimal cut-offs shown in Fig. 4, the predictive value of a positive test was 95%, the post-test likelihood of no disease following a negative test was 80%, whilst the post-test likelihood of disease following a negative test was thus only 20%. In those osteoporosis patients who had experienced a previous fracture, the predictive value of a positive test was similar at 92%, with the post-test likelihood of no disease following a n egative test rising to 100%. These results for cathepsin Z mRNA as a single biomarker are comparable to a peripheral blood biomarker panel consisting of 10 mRNAs including cathepsin Z mRNA for diagnosis of ischaemic stroke^19^. Furthermore, the observation that subjects with osteopenia also showed a significant increase in cathepsin Z mRNA compared to non-osteoporotic controls, strongly suggests that if replicated in a larger study, the cathepsin Z mRNA in patients’ PBMC preparations could form the basis of a test for osteoporosis, which could aid in the detection of osteoporosis before a critical and expensive fragility fracture occurs.

## Materials and Methods

### Clinical samples

The study conformed to the principles of the Helsinki Declaration and was carried out under ethical approval from the England Health Research Authority National Research Ethics Service Committee, East of England-Essex [REC reference 15/EE/0051] Ethics Committee. The inclusion and exclusion criteria for this study were as described previously^20^. Informed consent was obtained from all participants prior to sample collection. Participants were recruited from referrals to the Nuclear Medicine Department for a bone density scan at the Royal Liverpool University Hospital. In accordance with WHO guidelines on the diagnosis of osteopenia and osteoporosis, *T* scores of left femoral neck and lumbar spine (L2–4) were used.

### Isolation of peripheral blood mononuclear cells (PBMC)

Blood samples from participants were drawn into 9 mL S-Monovette® K3E, Potassium EDTA tubes by phlebotomists following a standard Safety Protocol. After centrifuging at 2,500 g (Thermo Heraeus Multifuge 3SRT centrifuge) for 30 min at room temperature, the buffy coat was transferred to a  15 mL centrifuge tube and diluted with an equal volume of Dulbecco’s Phosphate Buffered Saline (DPBS). 3 mL of Ficoll-premium (1.077 g/mL) solution was added into the bottom of a Greiner Bio-One 10 mL Leucosep® tube and centrifuged for 1 min at 900 g, to descend the porous barrier. Around 6 mL of DPBS-diluted blood samples were carefully pipetted down the side of the Leucosep® tube held at 45 degrees and the tube was centrifuged at 900 g at room temperature for 30 min. The interface layer containing the PBMCs was then collected into a new sterile 15 mL tube, and washed with DPBS, followed by centrifuging at 1,000 g at room temperature for 5 min to discard the supernatant. Cell pellets were then suspended with 10 mL of cold ammonium-chloride-potassium lysing buffer, and incubated at 4 °C for 1 min, followed by centrifuging at 2,500 g for 5 min at 4 °C. The pellets were washed twice using 10 mL DPBS, centrifugation at 2,500 g at room temperature for 5 min. Finally, the cell pellets were suspended with 0.5 mL DPBS, and transferred into 1.5 ml tubes and centrifuged at 14,000 g at 4 °C for 2 min to form a tight cell pellet, which was stored at −80 °C until required for further analysis.

### Isolation and purification of total RNAs

Total RNA from human PBMC cells was extracted using a combination of TRIzol reagent and PureLinkRNA mini kit (Thermo Fisher, UK), according to the manufactures’ recommendations. The resulting purified RNAs were eluted in 30 μL of RNase-free water and stored frozen at −80 °C until used. RNA concentrations and purity were measured using a Thermo Scientific NanoDrop™ 2000 spectrophotometer.

### Quantitation of mRNA levels using real-time quantitative PCR (RT-qPCR)

Reverse transcription and RT-qPCR reactions were carried out using RT-First Strand kit, RT-qPCR Primer Assays and SYBR Green PCR kit (Qiagen, UK), according to the manufacture’s recommendations. Briefly, 500 ng of purified RNA was reverse transcribed in 20 μL reactions using an RT-PCR kit (Qiagen). A separate reaction, in which RNase-free water replaced reverse transcriptase, provided a no-DNA control for subsequent RT-qPCR. Reverse transcription reactions were diluted 1:20 with RNase-free water prior to RT-qPCR amplification. RT-qPCR analyses were conducted in duplicate in 10 µL volume using RT-qPCR primer assays for human cathepsin Z or glyceraldehyde-3-phosphate dehydrogenase (GPDH) and SYBR Green PCR kit (Qiagen, UK) using a Roche LightCycler 96 Real-Time PCR system (Roche, UK). The no-reverse transcriptase control sample was included for each RT-qPCR amplification. All primers used for RT-qPCR in this study are listed in Supplemental Table S1. The relative levels of each RNA were determined from the Ct value after normalization with control molecule GPDH using the 2^−ΔΔCT^ method^21^. All Ct values obtained from RT-qPCR greater than 35 were considered to be below the detection level of the reaction.

### Statistical analysis

RT-qPCR results were statistically analysed as described previously^20^. Other statistical analyses were carried out using Stats Direct 3 software (Altrincham, Cheshire). *P* values of < 0.05 were considered significant.

## Supplementary information

Supplementary information
