# Supplementary material for: Cathepsin Z as a novel potential biomarker for osteoporosis
Source: Sci Rep. 2019 Jul 5;9:9752. doi: 10.1038/s41598-019-46068-0 (PMC6611782; doi:10.1038/s41598-019-46068-0)
Supplement: Supplementary file 1 — Supplementary information [file 41598_2019_46068_MOESM1_ESM.docx]

# Cathepsin Z as a novel potential biomarker for osteoporosis

Ayed A. Dera^1,5^, Lakshminarayan Ranganath^2^, Roger Barraclough^3^, Sobhan Vinjamuri^4^, Sandra Hamill^4^, Dong L. Barraclough^1*^

**Supplementary Figure S1. The relationship between cathepsin Z mRNA levels in PBMCs and chronic inflammation.** Panels a and b: box and whisker plots show that the levels of cathepsin Z mRNA are not significantly different between PBMCs from all the osteopenia and osteoporosis patients, without or with chronic inflammatory disorders (Panel a, 95% CI = -0.180 to 0.134, *P* = 0.774, Student’s t test) or PBMCs from the female osteopenia and osteoporosis patients over the age of 50 (Panel b, 95% CI = -0.244 to 0.157, *P* = 0.666, Student’s t-test). On each box and whisker plot, the black diamond shows the median value, the cross shows the mean value, white and black circles denote outliers of 1.5 times and 3 times the interquartile range, respectively.


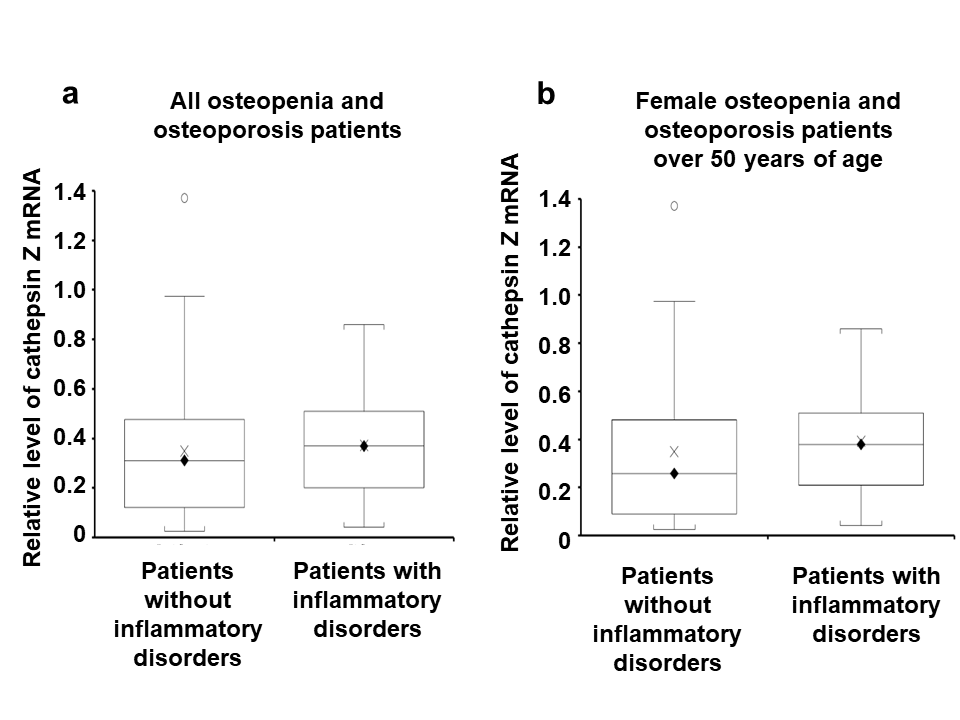


**Supplementary Figure S2**. **Association with osteoporosis of interferon-induced protein 16 (IFI16) mRNA levels in peripheral blood mononuclear cells.** Panels a and b: box and whisker plots show that the levels of mRNA for interferon-induced protein 16 (IFI16) are not significantly different between PBMCs from non-osteoporotic controls, osteopenia or osteoporosis patients when all participants (Panel a, one-way ANOVA (F(2,84) = 2.05, *P* = 0.135) or female participants over the age of 50 (Panel b, one-way ANOVA (F(2,60) = 0.612), *p* = 0.546) were included. On each box and whisker plot, the black diamond shows the median value, the cross shows the mean value, white and black circles denote outliers of 1.5 times and 3 times the interquartile range, respectively.


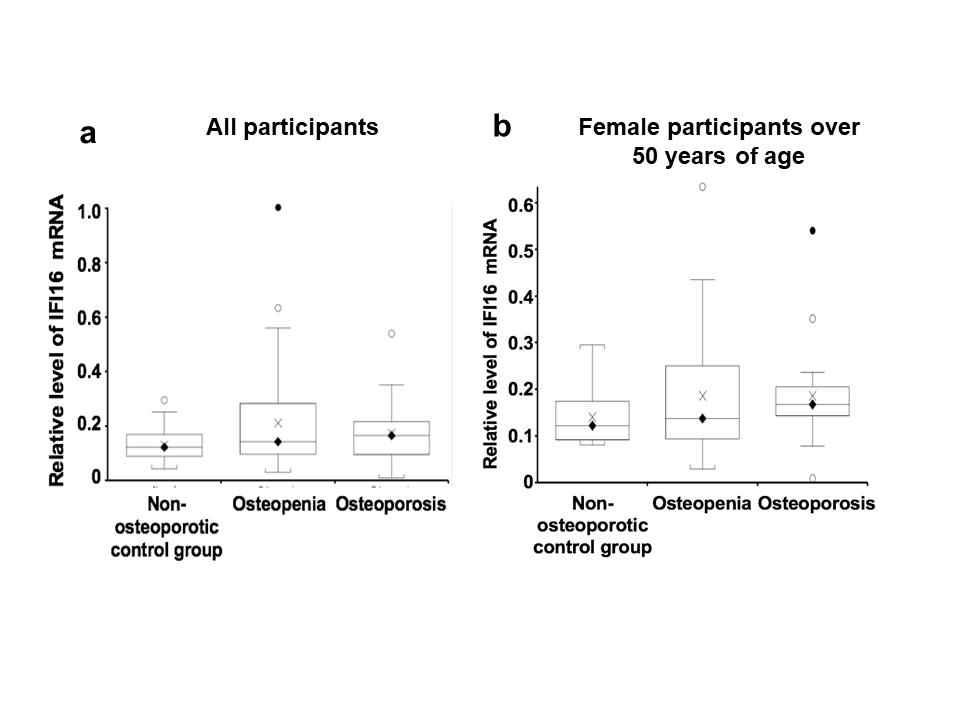


**Supplementary Figure S3. The relationship between interferon induced protein 16 (IFI16) mRNA levels in PBMCs and chronic inflammation.** Panels a and b: box and whisker plots show that the levels of IFI16 mRNA are not significantly different between PBMCs from all the osteopenia and osteoporosis patients, without or with chronic inflammatory disorders (Panel a, 95% CI = -0.032 to 0.097, *P* = 0.318, Student’s t test) or PBMCs from the female osteopenia and osteoporosis patients over the age of 50 (Panel b, 95% CI = -0.075 to 0.088, *P* = 0.876, Student’s t-test). On each box and whisker plot, the black diamond shows the median value, the cross shows the mean value, white and black circles denote outliers of 1.5 times and 3 times the interquartile range, respectively.


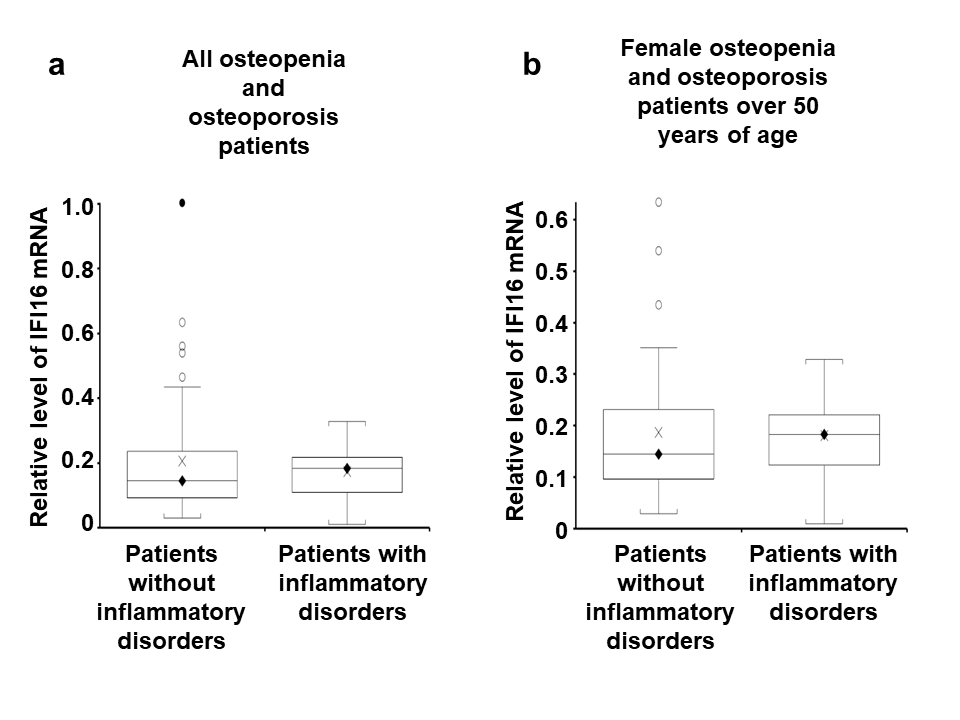


**Supplementary Table S1. A list of Qiagen primers used to amplify specific mRNAs by RT-qPCR.**

| **mRNA** | **Gene Symbol** | **Accession Number** | **Primer Sequence** | **Qiagen Catalogue** |
| --- | --- | --- | --- | --- |
| Cathepsin Z | CTSZ | NM_001336 | 5’ CTATAAGGATGGGAAGGGCGCC 3’ | PPH06136B |
| Glyceraldehyde-3-Phosphate Dehydrogenase | GAPDH | NM_001256799 | 5’ GGCGCTGCCAAGGCTGTGGGCA 3’ | PPH00150F |
